# Implementation Intentions on the Effect of Salt Intake among Hypertensive Women: A Pilot Study

**DOI:** 10.1155/2014/196410

**Published:** 2014-08-27

**Authors:** Rúbia de Freitas Agondi, Marilia Estevam Cornélio, Roberta Cunha Matheus Rodrigues, Maria-Cecilia Gallani

**Affiliations:** ^1^Faculty of Nursing, University of Campinas, Rua Tessália Vieira de Camargo 126, 13083-887 Campinas, SP, Brazil; ^2^Faculty of Nursing, Laval University, 1050 Avenue de la Médecine, Quebec, QC, Canada G1K 7P4

## Abstract

This experimental study was aimed at assessing the potential effect of a theory-driven intervention—implementation intentions—on reducing salt intake among hypertensive Brazilian women. Ninety-eight participants were randomly assigned to participate in an implementation intentions intervention aimed at promoting lower salt intake through decreased addition of salt and salty spices to meals (intervention group, *n* = 49; group, *n* = 49). Endpoints were assessed at baseline and at the 2-month follow-up. Primary endpoints were a self-reporting measure of salt intake given by salt addition to meals (discretionary salt + salty spices = total added salt) and the 24 h urinary-sodium excretion. Secondary endpoints included intention, self-efficacy, and habit related to adding salt to meals. Patients in the intervention group showed a significant reduction in salt intake as assessed by 24 h urinary-sodium excretion. A significant reduction in the measure of habit was observed for both groups. No differences were observed for intention and self-efficacy. The results of this pilot study suggest the efficacy of planning strategies to help hypertensive women reduce their salt intake.

## 1. Introduction

Hypertension has a high incidence and prevalence worldwide and is considered as a major risk factor for stroke, heart failure, ischemic heart disease, and chronic renal failure [1–4].

Hypertension is known as multifactorial disease and high sodium intake is recognized as a key factor for increased blood pressure in both healthy and hypertensive subjects [2, 3]. In fact, experimental [5] and clinical [6, 7] studies, reinforced by systematic reviews [8–10], have shown the significant, direct link between high sodium intake and increased blood-pressure levels and, consequently, cardiovascular risk. Conversely, a reduction in salt intake has been shown to result in a significant decrease in blood pressure among both normotensive and hypertensive individuals [5–10].

Thus, reducing dietary sodium intake has been recognized as an important nonpharmacological intervention to prevent and control hypertension. While guidelines for the treatment of hypertension have recommended a daily salt intake of approximately 3.7 g/day (1500 mg of sodium) [11, 12], salt intake remains high worldwide (9 to 12 g/day) [13–17]. Past studies conducted in Brazil [15, 16] pointed to high salt intake among hypertensive patients, ranging from 12.5 to 15.3 g/salt/day estimated by self-reporting methods and from 12.2 to 13.5 g/salt/day estimated by 24-hour urinary-sodium excretion. In both studies, the major source of total salt intake was discretionary salt, that is, the salt added during and after meal preparation, accounting for approximately 7.5 g/day of daily salt intake.

Cornélio et al. [16] used an extended version of the theory of planned behavior (TPB) [18, 19] in an attempt to understand the factors underlying salt intake among these hypertensive patients. Like other models in social psychology, TPB views motivation (intention) as the main determinant of behavior [18–22]. In Cornelio's study, however, although intention emerged as the main determinant of adding salt during meal preparation, it explained 22% of the behavior variance, pointing to a gap in the intention-behavior relationship. The literature has described this gap for other behaviors, attributing it mainly to the individuals that, while exhibiting positive intentions, fail to translate them into an action [23, 24].

Implementation intention has been used as an intervention strategy aimed at assisting people to translate their positive intentions into actions. This strategy consists of a mental simulation, linking situational cues and behavioral responses to specific future situations, specifying when, how, and where a particular action will take place. Thus, spontaneous reactions can be replaced with plans previously formulated. The action plan is a tool for developing self-regulatory skills to assist behavioral change, bringing to individual's consciousness the linkage between the situations of future realization of behavior and the possible behavioral response [24, 25]. Furthermore, coping plans are elaborated to overcome risk situations or barriers that might negatively influence the accomplishment of the behavior [26]. This cognitive planning represents a mental link between hazard anticipation to noncompletion of the planned behavior and possible coping responses [26, 27]. Thus, this strategy is of interest in empowering people to act on their intentions, even in situations in which barriers or obstacles change the action or when counterintentional behaviors are evoked, “protecting” their good intentions [26, 27].

Several studies have demonstrated the effectiveness of the implementation intentions strategy on the adoption of healthier nutritional behaviors, such as increasing fruit and vegetable consumption [28–31], reducing saturated-fat-food intake [32–34], and using weight-loss products [35].

To the best of our knowledge, specifically using implementation intentions to reduce salt intake has not been described. Thus, considering the need to develop and assess different interventions to reverse the problem of high salt intake mainly among hypertensive patients, this pilot study aimed at assessing the potential effectiveness of implementation intentions on reducing salt addition and the use of salty spices during and after meal preparation among hypertensive patients.

## 2. Methodology

### 2.1. Study Design and Procedure

This pilot study used a randomized controlled trial design. Participants were recruited from three health services: two outpatient clinics at a university hospital and one public primary-care unit, both located in a large city in southeastern Brazil. Potential participants were screened for eligibility based on the following inclusion criteria: (1) female; (2) responsible for her own meal preparation at home; (3) age ≥ 18 years; and (4) length of hypertension diagnosis ≥ six months. Men were excluded from the study because, in the target population, women are the main meal preparers in the home. Out of the 112 enrolled subjects, one was excluded for cognitive impairment limiting her comprehension of the questionnaires and 13 discontinued (6 decided to drop out of the study and 7 were lost to follow-up), resulting in a final sample of 98 women. The sample completing the study was compared with the dropout for the sociodemographic data and use of antihypertensive medications. There were no differences between them.

Data collection was performed in two steps (*T*_0_ and *T*_2_) within an interval of about two months from July 2010 to March 2011. Intervention was applied between these periods, as described.

At *T*_0_ (baseline), patients were invited to participate in the study in person when coming to the outpatient clinic at the regular time or by telephone. After obtaining their written informed consent, we collected the data through a semistructured interview: sociodemographic data (age, schooling, monthly income, marital, and employment statuses), self-reported measures of salt intake (discretionary salt and salty-spice intake), and psychosocial variables (intention, habit, and self-efficacy). At that time, participants were instructed how to collect urine for the 24 h urinary-sodium-excretion analysis and to return the urine sample one week later. Clinical data were obtained by chart analysis (length of hypertension diagnosis, antihypertensive medications in use) and physical evaluation (blood pressure and body mass index). Blood-pressure measurements followed the recommendations of the* Seventh Report of the National Committee on Prevention, Detection, Evaluation, and Treatment of High Blood Pressure* [36].

Once baseline data had been collected, the patients were randomized to the intervention (IG) or control (CG) groups according to a random-sequence list generated by SAS software (version 9.1.3, SAS Institute Inc., Cary NC, USA, 2002-2003).

#### 2.1.1. Intervention

Right after the randomization, IG patients received a letter containing information on the benefits of reducing dietary salt intake as a motivational strategy associated with implementation intentions intervention. Thus, one week later, IG patients returned for the intervention. The intervention was conducted in two in-person sessions intercalated with two telephone calls for reinforcement. In the first session, participants were asked to indicate up to three action plans on* when, where*, and* how *they thought they could reduce the salt added to food preparation during the next two months. Then, the women were asked to indicate* obstacles* or* barriers* that could interfere with the implementation of the plans they had proposed and to the strategies for* overcoming them* (*if … then … *[24]). The researcher made two copies of both plans: one for the researcher and the other for the participant [17]. The women were asked to repeat aloud the plans and instructed to put the plans in a visible and strategic place at home. Fifteen days later, the plans developed at *T*_0_ were reinforced by telephone call.

At *T*_1_ (40 days after *T*_0_), the IG patients returned for another in-person session to reassess and reinforce the plans developed at *T*_0_. A second telephone call for plans reinforcement was carried fifteen days later.

#### 2.1.2. Usual Care

Both groups received usual care from the health team, which included medical and nursing consultations, general counseling about pharmacological and nonpharmacological treatment, and treatment optimization. The usual care was provided throughout follow-up.

**Figure 1:**
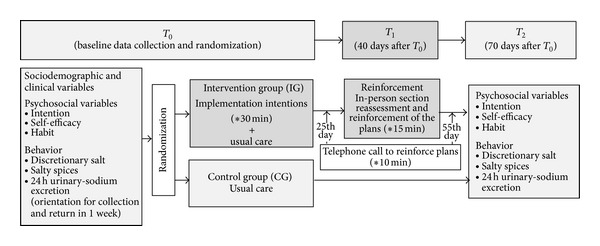
Data collection and intervention procedure.

At *T*_2_ (about two months after *T*_0_), IG and CG participants returned for the final measurements of psychosocial and behavioral variables. At the end of the study, all the participants received a “certificate” stating their participation in the research, a cooking measure equivalent to 4 g/salt, and a manual with low-salt recipes. An independent researcher trained in data collection and blinded to the randomization performed data collection at *T*_2_.  Figure 1 illustrates the data-collection and intervention procedure.

### 2.2. Measures

#### 2.2.1. Behavior

Salt intake was assessed by self-reported and biological measures. All the self-reported measureswere previously validated [15, 16].

*Total Added Salt.* This consisted in the sum of the values of estimated discretionary salt and salty spices, providing an estimate of the total amount of salt added to meals. The partial correlation (adjusting for age, schooling, and family income; *n* = 94) between total added salt and the 24 h urinary-sodium excretion was *r* = 0.25 (*P* = 0.014).

 
*Discretionary Salt.* This was based on previous studies [15, 16]. Patients were asked to rate their usual monthly amount of salt use (based on fractions of 1 kg salt packages) and the number of persons per household that had at least 5 meals per week at home, in order to correct the salt consumption per person. This was considered the reference (1 gram of salt = 400 mg of sodium) to obtain the monthly intake and, afterwards, the daily consumption in milligrams of sodium per person [2].
 
*Salty Spices.* This quantified the portion and frequency of monthly consumption of industrialized salty spices as tablets or packets. Subsequently, this value was adjusted by the number of persons in the household and their respective weekly number of meals at home in order to estimate the daily sodium intake resulting from salty spices.

*24 h Urinary-Sodium Excretion.* The women were carefully instructed to collect the total urinary volume during the 24 h period and to maintain their normal water intake. Sodium excretion was measured by spectrophotometry and converted to mEq/L [37]. Sodium intake was estimated, assuming that 1 mEq sodium equals approximately 0.058 g of salt intake [38].

#### 2.2.2. Psychosocial Variables

*Intention.* This variable was assessed with six items using a five-point Likert-type scale (e.g.,* I intend* to add less than 4 g of salt a day when cooking my meals:* definitely not … definitely yes*). The final score of intention was the arithmetic mean of the six items. Higher scores pointed to more positive intention [16]; in this study, the Cronbach's alpha coefficients for this scale were 0.91 at *T*_0_ and 0.89 at *T*_2_.

*Self-Efficacy.* Perceived self-efficacy was assessed with three questions on a five-point Likert-type scale (e.g.,* I trust in my *ability to add less than one teaspoon of salt a day when cooking all my meals:* definitely not … definitely yes*). The final score of self-efficacy was the arithmetic mean of the three items. Higher scores indicated high levels of perceived self-efficacy [16]; in this study, the Cronbach's alpha coefficients for this scale were 0.91 at *T*_0_ and 0.91 at *T*_2_.

*Habit.* The habit of adding more than one teaspoon salt/day/person during meal preparation was assessed with ten items on a five-point Likert-type scale (e.g., adding more than one teaspoon of salt a day when cooking all my meals is something* I do frequently*:* definitely not … definitely yes*). The final habit score was the arithmetic mean of the ten items. Higher scores indicated a stronger habit of adding more than 4 g/day of salt when cooking meals [16]; in this study, the Cronbach's alpha coefficients for this scale were 0.94 at *T*_0_ and 0.88 at *T*_2_.

### 2.3. Data Analysis

Data were presented as means (standard deviation) and 95% confidence intervals. The intervention's effect was assessed with repeated-measures ANOVA tests. A significance of *P* < 0.05 was adopted. All analyses were conducted with SAS software (version 9.1.3, SAS Institute Inc., Cary NC, USA, 2002-2003). Chi-squared and Mann-Whitney tests were used to test differences between sociodemographic and clinical measures between the groups.

### 2.4. Ethical Aspects

The local ethics committees approved the study and all enrolled patients signed an informed consent form.

## 3. Results

### 3.1. Descriptive Data

Table 1 provides the sociodemographic and clinical features. The subjects in the total sample were, on average, 60 years old with 4.1 years of schooling, lived with a partner (60.7%), and were not in the labor force (50.1%). The majority of the participants were overweight or obese, with a mean body mass index of 31.6 kg · m^2^. The mean length of hypertension diagnosis was 15.7 years; blood-pressure values were 140 and 82.2 mmHg for systolic and diastolic. On average, the participants took 2.6 classes of antihypertensive drugs; rennin inhibitors (angiotensin-converting enzyme inhibitors or angiotensin II receptor blockers) and diuretics were the most commonly used (83.8% and 69.4%, resp.).

Table 2 presents descriptive statistics on behavior measures and psychosocial variables at baseline and at the 2-month follow-up for CG and IG participants. At baseline, both groups exhibited a high sodium intake evidenced by self-reported and objective measures. The sodium intake estimated by the 24 h urinary sodium was higher than that estimated by the sum of the self-reported measures. This difference can be accounted for by the fact that “total added salt” does not consider all sources of salt intake, but only the salt and salty spices added to meals.

The scores of the psychosocial variables pointed to a group motivated to limit the addition of salt to meals with a perception of self-efficacy to do so, while recognizing the habit of adding more than 4 g salt/day during meal preparation.

### 3.2. Results of Intervention

**Figure 2:**
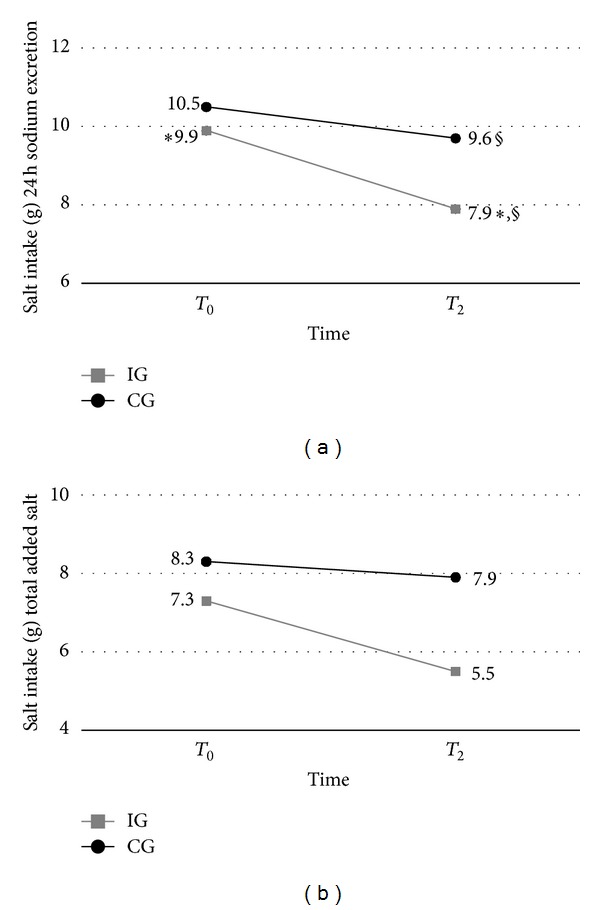
Effect of implementation intentions interventions on salt intake estimated by 24 h urinary sodium excretion and total added salt. *P = 0.0011; ^§^ *P* = 0.0395. Repeated-measures ANOVA.

The models for repeated-measures ANOVA analysis showed that, at the 2-month follow-up (*T*_2_), there was a significant reduction (*P* = 0.001) in the 24 h urinary-sodium excretion; the measurement at *T*_2_ was significantly lower in the IG group (*P* = 0.039) (Figure 2). Despite the reduction indicated in the self-reported measures, salt intake was not significantly lower (total added salt, *P* = 0.0504). The CG evidenced no significant differences in salt consumption during the follow-up. Thus, the data suggest that the strategy of implementation intentions was effective in reducing overall salt intake.

**Figure 3:**
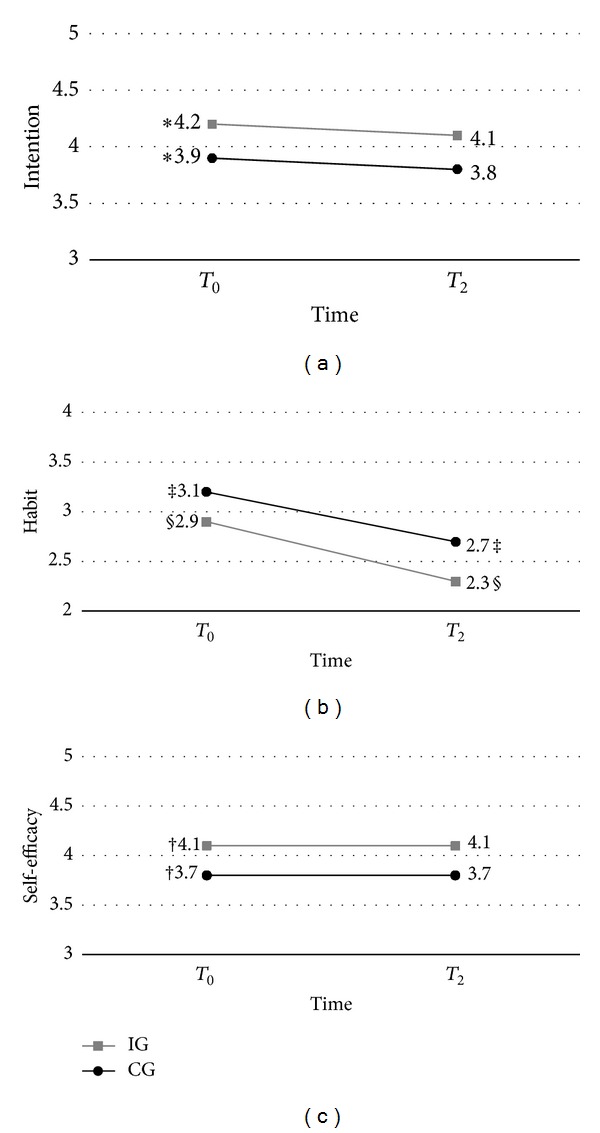
Effect of implementation intentions interventions on psychosocial variables intention, self-efficacy, and habit. *P = 0.002; ^‡, §^ *P* < 0.0001; ^†^ *P* = 0.001. Repeated-measures ANOVA.

The intervention effect on the psychosocial variables potentially involved in the process of behavior change was also analyzed. We observed that the intervention had no impact on intention and self-efficacy, as no differences in these variables were observed between *T*_0_ and *T*_2_ in either group. Nonetheless, at *T*_2_, a significant reduction in the measure of habit was observed in both groups, with the score for the IG being significantly lower than that for the CG. This result suggests that intervention only had an effect on the psychosocial variable habit (Figure 3).

## 4. Discussion

The results of this study indicate that the women formulating plans on how to implement the behavior and how to overcome the potential barriers to action showed a significant 2 g reduction in salt intake, evidenced by the 24 h urinary-sodium analysis. The self-reported measure of salt added to the meals also exhibited a reduction, but it was within the limits of statistical significance. These results support recent findings in the literature demonstrating the effectiveness of implementation intentions strategies in helping individuals adopt different nutritional behaviors, such as eating healthier foods like fruit and vegetables [28–31] and refraining from unhealthy foods, such as high-fat items [32, 33]. These data are reinforced by a recent meta-analysis indicating that implementation intentions strategies seem to be more effective in promoting the consumption of healthy foods and reducing the consumption of unhealthy foods [39]. No data were available on using this strategy to reduce salt intake, reinforcing the importance and uniqueness of our study.

It is important to note that, notwithstanding the significant reduction in total salt intake as evidenced by 24 h urinary-sodium excretion (the only measure in this study reflecting total/overall salt intake), the IG salt intake at *T*_2_ was still higher than the values recommended for hypertensive subjects. This result was nonetheless expected, considering the short follow-up period. Longer exposure to the intervention would be necessary to yield larger, more sustained reductions in salt intake. In fact, the gradual reduction of this nutrient is indeed an attractive approach. Firstly, it helps the individual adapt taste, preserving the pleasure of eating associated with salty foods [40]. The assimilation of the use of natural alternatives for seasoning—such as natural spices—as mentioned by the participants in their planning [17], seems to be a useful strategy to help adapt taste to a diet with a lower salt content. Secondly, as evidenced by a recent systematic review [10], long-term (≥4 weeks), modest salt reduction resulted in lower blood pressure with slight increases in the physiological activity of the renin-angiotensin-aldosterone system, without significant changes in the lipid profile. This contrasts with the meta-analyses that included a large number of short-term trials with a large change in salt intake. In this review, a major impact on salt reduction was related to adverse effects on hormones and lipids that could mitigate the benefits yielded by lower blood pressure [10].

Regarding psychosocial variables, as expected, no changes were observed for intention in the follow-up. Our data reinforce the findings of past studies that the implementation intentions strategies do not modify the score of intentions but are effective in helping implement good intentions on actions [41].

Self-efficacy can be involved in the success of behavioral change in response to the implementation intentions strategy. The formulation of the plans to overcome the barriers can positively affect the perception regarding the ability to execute the behavior. Our data, however, do not support this assumption, since there was no significant difference in the scores for this variable in response to the intervention.

Conversely, there was significant reduction in the scores for habit related to the addition of more than 4 g/salt/day during meal preparation. In the IG, this could be explained by the nature of the action plan drawn up, which included plans to quantify the amount of added salt, which can change the automatism related to the behavior. Interestingly, authors have remarked that implementation intentions can be helpful in breaking down habits. The formulation of counterhabitual plans would reduce the relative strength of mental links between a habitual behavior and the critical situation triggering it [42]. Thus, in the context of habitual behaviors, the use of implementation intentions might assist in attaining the target behavior by disrupting habitual responses [24, 42, 43].

It is noteworthy that the control group showed lower habit scores at *T*_2_ as well. It is possible that cognitive pathways activated by answers to the questionnaire acted as cues to disrupt the behavior automatism, bringing it to a more conscious level, but less effectively than implementation intentions [44].

This study has some limitations. Due to its nature as a pilot study, we had a relatively small number of subjects, which may explain the nonsignificance of the intervention effect on the self-reported measures of salt intake. The short follow-up period is another limitation. The literature contains conflicting findings about the temporal stability of the effects of implementation intentions strategies. Authors argue that the effect remains even for some time after the end of intervention [24, 26, 27, 41]. There is, however, evidence that the effect of implementation intentions strategies is temporally limited for behaviors requiring significant effort to initiate and sustain and for which the benefits of behavioral change appear only on the medium or long term [35]. Thus, medium- and long-term follow-up studies are needed to assess if the reduction in salt intake should be maintained or even lowered further to attain intake values nearer to those recommended in the guidelines. This study recruited only women, due to the nature of the target behavior. Other strategies must permit the inclusion of male patients. The approach of dyads can be an alternative, so more complex designs would be needed to verify intervention effectiveness. Finally, in addition to patient recruitment from different health-care settings, the intervention was applied by a single nurse. This might have resulted in a bias in the intervention results related to personal delivery. Thus, further studies could consider a multicentric approach, with the intervention being applied by different well-trained nurses.

On the other hand, this pilot study's strengths should be pointed. It was a RCT study strictly following the recommendations for good practices in clinical trials [45]. As a final point, the uniqueness of the study's originality must be pointed out. Indeed, it was the first to use the strategy of implementation intentions to reduce salt intake among hypertensive patients and in a clinical context.

The use of theory-based intervention is important to health professionals helping patients change behaviors. Implementation intentions strategies are fast and simple to use and can help people change their unhealthy behaviors.

## 5. Conclusion

The use of the strategy of implementation intentions to reduce additions of salt and salty spices to meals appears to point to effectiveness in reducing dietary salt intake among hypertensive women. Studies with larger samples and longer follow-ups are needed to generalize the results and to assess the medium- and long-term effects of the intervention on maintaining and further reducing salt intake.

## Figures and Tables

**Table 1:** Sociodemographic and clinical profile of the control group and intervention group at baseline.

	Intervention group (*n* = 55)	Control group (*n* = 57)
	Mean (SD)	Mean (SD)
Age (in years)	59 (8.0)	61.0 (11.0)
Schooling (in years)	4.0 (3.0)	4.0 (3.0)
Family monthly income (US$)	728.5 (505.1)	795.0 (467.4)
Diagnosis length (in years)	16.0 (11.0)	16.0 (12.0)
Systolic blood pressure (mmHg)	146.0 (24.0)	138.0 (22.0)
Diastolic blood pressure (mmHg)	85.0 (15.0)∗	79.0 (12.0)∗
Body mass index	31.7 (6.2)	31.5 (5.1)
Number of antihypertensive drugs in use	2.7 (1.1)	2.5 (1.1)

	*n*	*n*

Marital status		
With partner	33	35
Without partner	22	22
Work status		
Active	8	6
Inactive	23	33
Housewife	24	18
Antihypertensive drugs		
Renin inhibitor	50^†^	43^†^
Diuretics	37	40
Ca-blocker	23	32
Beta-blocker	26	20
Alpha-blocker	9	3
Direct arterial dilators	2	2

*P < 0.05 (Mann-Whitney test); ^†^ *P* < 0.05 (chi-square test); SD: standard deviation; Ca-blocker: calcium-channel blocker.

**Table 2:** Measures of salt intake and psychosocial variables for the IG and CG at baseline and at the 2-month follow-up.

	Intervention group (IG) (*n* = 49)				Control group (CG) (*n* = 49)			
	*T*_0_		*T*_2_		*T*_0_		*T*_2_	
	Mean (SD)	95% CI	Mean (SD)	95% CI	Mean (SD)	95% CI	Mean (SD)	95% CI
Salt Intake								
Total salt (in grams)	7.3 (5.0)	5.9–8.8	**5.5 (3.2)**	4.6–6.5	8.3 (5.2)	6.6–9.7	**7.9 (6.8)**	5.5–9.3
Discretionary salt (in grams)	6.7 (4.9)	5.4–8.3	5.3 (3.1)	4.5–6.3	7.6 (4.8)	6.1–8.9	7.3 (5.9)	5.3–8.7
Salty spices (in grams)	0.5 (0.7)	0.3–0.7	0.1 (0.4)	0.03–0.3	0.7 (1.3)	0.3–1.1	0.5 (1.5)	0.1–0.7
Salt: urinary sodium (in grams)	9.9 (4.1)	8.8–11.1	**7.9 (3.3)**	7.0–8.9	10.5 (4.2)	9.4–11.8	**9.6 (2.9)**	8.7–10.5

Psychosocial variables								
Intention	4.2 (0.5)	4.1–4.4	4.1 (0.4)	4.1–4.3	3.9 (0.7)	3.7–4.1	3.8 (0.7)	3.7–4.1
Self-efficacy	4.1 (0.6)	4.0–4.3	4.1 (0.5)	3.9–4.2	3.7 (0.8)	3.6–4.0	3.7 (0.8)	3.6–4.0
Habit	2.9 (1.0)	2.7–3.3	2.3 (0.5)	2.2–2.4	3.1 (0.9)	2.8–3.3	2.7 (0.7)	2.4–2.8

SD: standard deviation; 95% CI: 95% confidence interval.
